# SARS-CoV-2 vaccination diversifies the CD4+ spike-reactive T cell repertoire in patients with prior SARS-CoV-2 infection

**DOI:** 10.1016/j.ebiom.2022.104048

**Published:** 2022-05-06

**Authors:** Arbor G. Dykema, Boyang Zhang, Bezawit A. Woldemeskel, Caroline C. Garliss, Rufiaat Rashid, Timothy Westlake, Li Zhang, Jiajia Zhang, Laurene S. Cheung, Justina X. Caushi, Drew M. Pardoll, Andrea L. Cox, Hongkai Ji, Kellie N. Smith, Joel N. Blankson

**Affiliations:** a Bloomberg∼Kimmel Institute for Cancer Immunotherapy, Johns Hopkins University, Baltimore, MD, USA; b Sidney Kimmel Comprehensive Cancer Center, Johns Hopkins University, Baltimore, MD, USA; c Department of Biostatistics, Bloomberg School of Public Health, Johns Hopkins University, Baltimore, MD, USA; d Department of Medicine, School of Medicine, Johns Hopkins University, Baltimore, MD, USA

**Keywords:** SARS-CoV-2, Coronavirus, COVID-19, CD4+ T cells, mRNA vaccination

## Abstract

**Background:**

COVID-19 mRNA vaccines elicit strong T and B cell responses to the SARS-CoV-2 spike glycoprotein in both SARS-CoV-2 naïve and experienced patients. However, it is unknown whether the post-vaccine CD4+ T cell responses seen in patients with a history of COVID-19 are due to restimulation of T cell clonotypes that were first activated during natural infection or if they are the result of new clones activated by the vaccine.

**Methods:**

To address this question, we analyzed the SARS-CoV-2 spike glycoprotein-specific CD4+ T cell receptor repertoire before and after vaccination in 10 COVID-19 convalescent patients and 4 SARS-CoV-2 naïve healthy donor vaccine recipients. We used the viral Functional Expansion of Specific T cells (ViraFEST) assay to quantitatively identify specific SARS-CoV-2 and common cold coronavirus CD4+ T cell clonotypes post COVID-19 disease resolution and post mRNA SARS-CoV-2 vaccination.

**Findings:**

We found that while some preexisting T cell receptor clonotypes persisted, the post-vaccine repertoire consisted mainly of vaccine-induced clones and was largely distinct from the repertoire induced by natural infection. Vaccination-induced clones led to an overall maintenance of the total number of SARS-CoV-2 reactive clonotypes over time through expansion of novel clonotypes only stimulated through vaccination. Additionally, we demonstrated that the vaccine preferentially induces T cells that are only specific for SARS-CoV-2 antigens, rather than T cells that cross-recognize SARS-CoV-2/common cold coronaviruses.

**Interpretation:**

These data demonstrate that SARS-CoV-2 vaccination in patients with prior SARS-CoV-2 infection induces a new antigen-specific repertoire and sheds light on the differential immune responses induced by vaccination versus natural infection.

**Funding:**

Bloomberg∼Kimmel Institute for Cancer Immunotherapy, The Johns Hopkins University, The Bill and Melinda Gates Foundation, NCI U54CA260492, NIH.

**Research in context**

**Evidence before the study**

To design this study, we searched the scientific literature using PubMed to identify other studies that evaluated SARS-CoV-2 vaccination efficacy in patients with history of COVID-19. We used the search terms “SARS-CoV-2”, “vaccination”, “COVID-19 convalescent,” and identified many studies evaluating vaccination in both COVID-19 convalescent and healthy individuals receiving the SARS-CoV-2 mRNA vaccinations. However, when we included “T cell receptor sequencing” and “T cell clonotype” in our search we only identified one manuscript that used TCR-sequencing. Of note, this paper by Minervina et al. (10.1101/2021.07.12.21260227), exclusively looked at MHC I tetramers thereby limiting their study to CD8+ T cells.

**Added value of the study**

Our findings demonstrate that SARS-CoV-2 mRNA vaccination induces a novel repertoire of CD4+ T cells that specifically expanded to the SARS-CoV-2 spike glycoprotein in the context of vaccination, but not in natural infection. We show that COVID-19 convalescent patients mount robust CD4+ T cell responses to SARS-CoV-2 both following natural infection and after mRNA vaccination, however the exact clonotypes contributing to this response are largely divergent at the timepoint studied. Consistent with other reports, we show that T cell responses wane up to 6 months post infection or vaccination through contraction of reactive clonotypes in the peripheral blood. However, this study uniquely establishes the composition of SARS-CoV-2-reactive T cells (both infection-induced and vaccine-induced) at the resolution of specific TCR clonotypes. This study not only confirms the lifespan of SARS-CoV-2 TCR clonotypes but proves that vaccination can maintain the overall number of clones through induction of new clones, thereby diversifying the repertoire.

**Implications of all the available evidence**

Vaccination improves the duration of immune response in patients with prior history of COVID-19 by initiation of novel T cell clonotype reactivity. These data confirm the utility of vaccination in all people regardless of their COVID-19 history. Additionally, we have uniquely compiled a dataset of SARS-CoV-2-reactive TCR CDR3 sequences and characterized their responses to SARS-CoV-2 as well as their reactivity to other endemic human coronaviruses. This information can be used to query other publicly available datasets to assess global trends of SARS-CoV-2 repertoires and assess patient populations with waning immunity. It may also be used as a complementary analytic tool to antibody titers to compare and assess efficacy and dosing regimen of various vaccine formulations that may arise in the near future.

Alt-text: Unlabelled box

## Introduction

The Pfizer-BioNTech (BNT162b2) and Moderna (mRNA-1273) SARS-CoV-2 vaccines induce robust CD4+ T cell responses.^1^^,^^2^ However questions still remain regarding the necessity of vaccinating patients with “natural immunity” from prior SARS-CoV-2 infection.^3^ Vaccination with either of the mRNA vaccines results in transcription and translation of the SARS-CoV-2 full-length spike glycoprotein, thus allowing for a comprehensive immune response to a variety of antigenic spike-derived epitopes. It is unclear how immune responses primed by infection are affected by subsequent SARS-CoV-2 immunization. While serum antibody titers are typically used to assess levels and duration of SARS-CoV-2 immunity, CD4 helper T cell responses are the key determinants of immune memory, which ultimately determines the speed and magnitude of cytotoxic T cell and antibody responses upon re-exposure to the virus.

Prior studies have established the existence of CD4+ T cells that cross-recognize SARS-CoV-2 and endemic common cold coronaviruses (CCCs).^4^, ^5^, ^6^, ^7^, ^8^, ^9^, ^10^, ^11^, ^12^ These cross-reactive T cells could have major impact on immunologic memory in the present COVID-19 pandemic and in possible zoonotic coronavirus outbreaks in the future. Of importance, cross-reactive T cells have been shown to be of lower functional avidity for SARS-CoV-2 which may result in reactive T cells with reduced biologic relevance.^12^ Furthermore, endogenous T cell responses induced from SARS-CoV-2 infection protect against reinfection in macaques,^13^ but it is unknown whether vaccination of COVID-19 convalescent patients (CCPs) provides additional benefit or whether natural infection confers sufficient immune protection. Indeed, mRNA vaccination increases SARS-CoV-2 antibody titers in CCPs beyond what was achieved during natural infection,^14^^,^^15^ yet the T cell response to vaccination in these patients has not been studied at the clonal level. It is therefore necessary to assess the precise clonal nature of the vaccine-induced T cell repertoire and to understand changes in the SARS-CoV-2-reactive T cell repertoire after vaccination of previously infected individuals. In this study we evaluated the clonality and magnitude of SARS-CoV-2-reactive CD4+ T cells in CCPs after vaccination and compared post-vaccination immune responses to those following natural SARS-CoV-2 infection.

**Figure 1:**
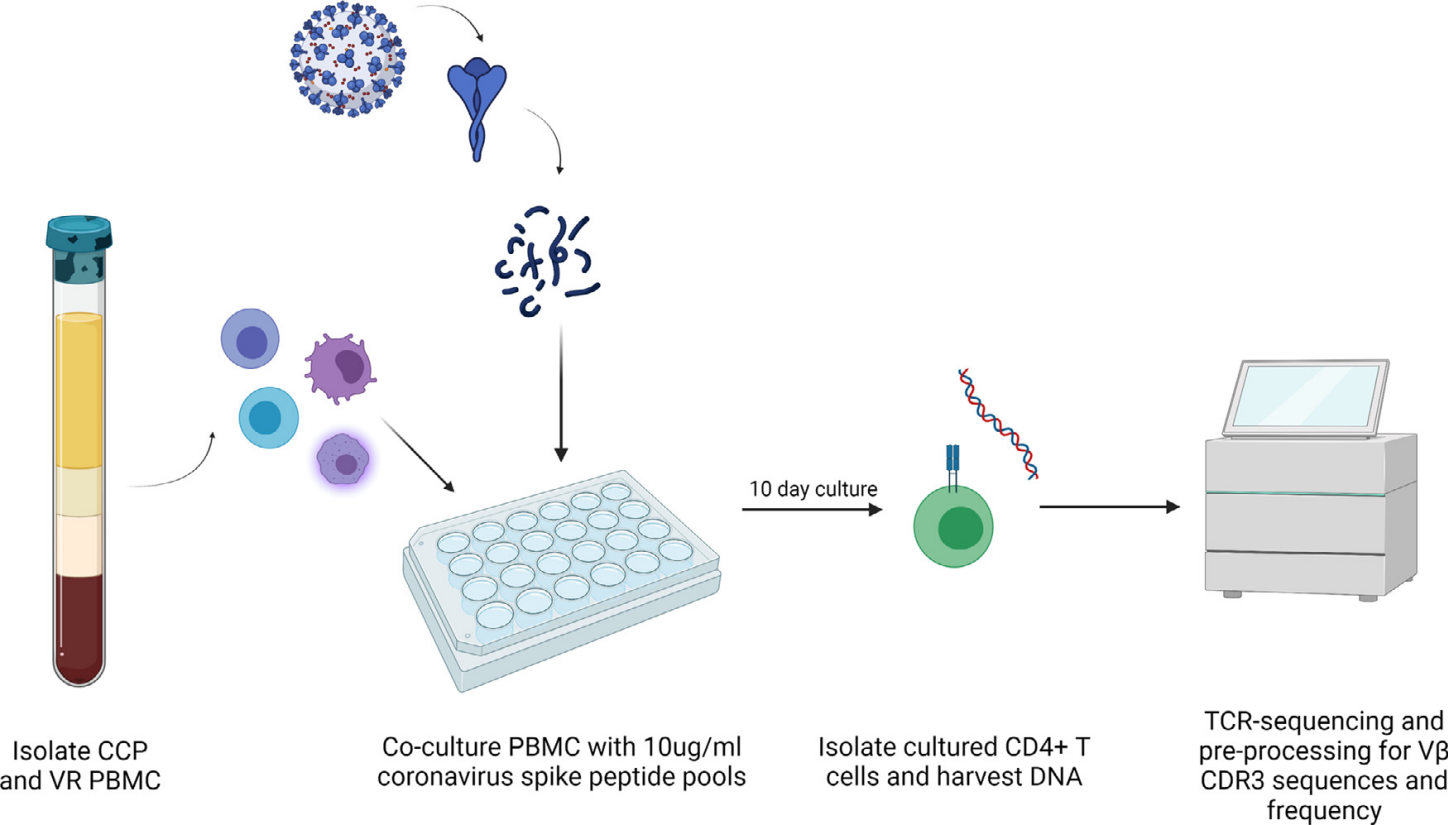
Coronavirus CD4 ViraFEST experimental schematic. Peripheral blood mononuclear cells (PBMC) were isolated from COVID-19 convalescent (CCP) and healthy donor vaccine recipients (VR). 2 million PBMC were stimulated with coronavirus spike peptide pools at a final concentration of 10ug/ml individual peptides in 24-well plates. After 10-days, CD4+ T cells were isolated via negative selection. DNA was isolated and TCR-sequencing was performed.

To specifically test SARS-CoV-2 reactivity at the CD4 TCR Vβ clonal level, we used the viral Functional Expansion of Specific T cells (ViraFEST) assay,^12^^,^^16^, ^17^, ^18^, ^19^, ^20^ which is a recently developed functional T cell assay that identifies antigen-specific T cell receptor (TCR) clonotypes (Figures 1, and S1, S2). Here, we use this assay to uncover a distinct SARS-CoV-2-specific repertoire that is induced by vaccination versus natural infection. Additionally, as compared to the standard ELISPOT or intracellular cytokine stimulation assays, ViraFEST uniquely assesses reactivity and repertoire on the clonal level, allowing us to examine whether the precise nature of the immune response after vaccination consists of cross-reactive or SARS-CoV-2 mono-reactive T cell clones. Our findings enhance our understanding of the vaccine-induced immune repertoire in people with a history of COVID-19 and demonstrate the potential to enhance the diversity of T cell responses to SARS-CoV-2 through vaccination, even in patients with residual immunity from natural infection.

## Methods

### Study participants and biospecimens

This study was conducted according to Declaration of Helsinki principles. COVID-19 convalescent patients (CCPs) refer to patients who tested positive for SARS-CoV-2 by nasal swab PCR test. Nine out of the ten patients had mild disease and 1 patient had severe disease. All 10 patients have since recovered from COVID-19. The date range of positive PCR test was from 03/2020 to 12/2020, prior to the United States emergence of the Alpha (B.1.1.7), Beta (B.1.351, B.1.351.2, B.1.351.3), and Delta (B.1.617.2, AY.1 – AY.12) SARS-CoV-2 variants. Healthy vaccine recipients (VRs) refer to peripheral blood mononuclear cell (PBMC) donors who received a mRNA SARS-CoV-2 vaccination without having prior infection with SARS-CoV-2 as documented by negative nucleocapsid antibodies pre- and post-vaccination. PBMC samples were taken prior to and after receiving two doses of either the Pfizer-BioNTech (BNT162b2; *n* = 8) or Moderna (mRNA-1273; *n* = 2) mRNA SARS-CoV-2 vaccination. There were six male and four female CCPs, two of whom were Hispanic. There were seven Caucasians, two Asians, and one multiracial individual. All the CCPs except CCP2 had mild disease and were not hospitalized. CCP2 has well controlled HIV on antiretroviral therapy and developed severe disease. Post-COVID samples were obtained from CCPs a median of 101 days after COVID-19 diagnosis (range 35 to 161). Post vaccine samples were taken from CCPs and VRs a median of 72 days after the second dose of the vaccine (range 14 to 241). CCP6 and CCP11 were sampled immediately prior to vaccination (Day -16 and -7, respectively). CCP6, CCP11, and CCP16 were sampled both early post vaccination (day +14) and late post vaccination (day +241, +227, and +161, respectively). *Ex-vivo* TCR-sequencing was performed on two participants (VR8 and VR10) from PBMC harvested pre- and post- boost vaccination. VR8 and VR10 received the booster shots 10 and 9 months after the second vaccine dose, respectively. VR8 had two Pfizer-BioNTech (BNT162b2) vaccinations prior to boost and VR10 had two Moderna (mRNA-1273) vaccinations prior to boost, both participants received a Moderna (mRNA-1273) mRNA SARS-CoV-2 vaccination as boost. All participants were enrolled according to protocols approved by the Johns Hopkins University IRB and provided written informed consent. PBMCs from each study participant were isolated from whole blood via Ficoll-Paque Plus gradient centrifugation and were viably cryopreserved at -140C or were used immediately in the FEST assay. Antibodies to the nucleocapsid protein were measured with the Bio-Rad Platelia SARS-CoV-2 Total Ab assay (Marnes-la-Coquette, France) and used to rule out natural infection with SARS-CoV-2 in the 4 VRs as previously described.^21^

### Identification of human coronavirus-specific T cells

The ViraFEST assay,^12^^,^^16^, ^17^, ^18^, ^19^, ^20^ identifies antigen-specific T cell receptor (TCR) clonotypes. As shown in Figure 1, the assay involves stimulating T cells with peptide pools to induce antigen-specific expansion followed by T cell receptor sequencing to identify antigen-specific T cell clonotypes (Figures 1**, and S1, S2**). For the experiments described here, overlapping peptide pools spanning the S protein of three common human coronaviruses (HCoV-NL63, HCoV-OC43, and HCoV-229E; BEI and jpt), as well as overlapping peptide pools spanning the S protein of SARS-CoV-2 (BEI and jpt) were used to stimulate CD4+ T cells in the ViraFEST assay as described previously,^22^ with minor modifications.^12^^,^^17^^,^^22^ 2 x 10^6^ PBMC were plated in culture medium (IMDM, 5% human AB serum, 10 IU/ml IL-2, 50 ug/mL gentamicin) with 10ug/ml of individual HCoV and SARS-CoV-2 peptide pools, a positive control CEFX Ultra SuperStim consisting of pooled CMV, EBV, and Flu MHC-II restricted epitopes (jpt, PM-CEFX-3), a negative control HIV-1 Nef peptide pool (NIH AIDS Reagents), or without peptide. Each assay condition was performed in triplicate unless otherwise noted. On days 3 and 7, half the media was replaced with fresh culture media containing IL-2 (final concentration of 10 IU/mL Il-2). On day 10, cells were harvested and CD4+ T cells were isolated using the EasySep CD4+ T cell isolation kit (STEMCELL, 17952). DNA was extracted from cultured CD4+ T cells using the Qiamp micro-DNA kit according to the manufacturer's instructions. TCRseq of DNA extracted from cultured CD4^+^ T cells was performed by the Johns Hopkins FEST and TCR Immunogenomics Core Facility (FTIC) using the Oncomine TCR Beta short-read assay (Illumina, Inc). Samples were pooled and sequenced on an Illumina iSeq 100 using unique dual indexes.

Data pre-processing was performed to eliminate non-productive TCR sequences and to align and trim the nucleotide sequences to obtain only the CDR3 region. Sequences not beginning with “C” or ending with “F” or “W” and having less than 7 amino acids in the CDR3 were eliminated. Resulting processed data files were uploaded to our publicly available MANAFEST analysis web app (www.stat-apps.onc.jhmi.edu) to bioinformatically identify antigen-specific T cell clonotypes. Clones were considered positive based on the following criteria: (1) significantly expanded in the culture of interest (in two of three replicate wells) compared to the reference culture (PBMC cultured with 10 IU/ml IL-2 and HIV-1 Nef pool or media without peptide for HIV+ donor CCP2) at a false discovery rate (FDR) less than the specified threshold (<0.05; default value), (2) significantly expanded in the culture wells of interest compared to every other culture well performed in tandem (FDR<0.05; default value), (3) have an odds ratio >5 (default value) and (4) having productive frequency of >0.1% in at least two of the three replicate wells. Cross-reactive clonotypes are defined by the same criteria but with significant expansion in SARS-CoV-2 S replicate wells as well as at least one of the additional CCC spike protein wells in replicate using statistical criteria established previously.^17^

### IFN-γ ELISPOT assay

SARS-Cov-2 peptides were obtained from BEI Resources and reconstituted with DMSO at a concentration of 10 mg/ml. The SARS-CoV-2 peptides are 12 mer, 13 mer, or 17 mer, with 10 amino acid overlaps. IFN-γ ELISPOT assays were performed as previously described.^7^ Peptides were diluted to 1 ug/ml in culture media. Stimulation with anti-CD3 antibody (Mabtech, 1 μg/mL) was used as a positive control for each study participant. Briefly, ELISPOT Pro and ELISPOT Plus kits with precoated plates were purchased from Mabtech. The wells were plated with PBMCs at 250,000 cells/well, and the cells were cultured for 20 h. ELISpot plates were read by a blinded independent investigator on an AID iSpot Spectrum using vendor-provided software that reported SFU/well. Each peptide was run in duplicate, and a peptide was only considered to be positive if the average stimulation index (fold change over untreated control) was above 3, and more than 20 SFU/106 cells were present. *Unrooted phylogenetic trees*

The non-redundant TCR sequences were defined by excluding the first 3 and last 3 amino acids of the TCR Vβ CDR3 region due to significant sequence overlap at the beginning and the end of the CDR3 sequence.^23^^,^^24^ The levenshtein distance between each pair of TCR sequences was calculated based on non-redundant TCRs, using the ‘stringdist’ R package.^25^ The TCR sequence homology pattern was visualized in an unrooted phylogenetic tree for each participant, where each leaf of the unrooted phylogenetic tree represented a TCR Vβ CDR3 sequence. The unrooted phylogenetic trees were generated using the ‘ape’ R package.^26^ All analyses were performed using R software, version 3.6.1.

### Statistics

Wilcoxon signed-rank test was performed to compare the number of CD4+ T cell clonotypes after vaccination to the number of clonotypes after natural infection (*p* = 0.4258). Wilcoxon signed-rank test was performed to compare the total number of unique mono- and cross-reactive clonotypes after vaccination for both CCPs and VRs (*p* = 0.002; *p* = 0.1250).

### Ethics

This study was approved by the IRB of Johns Hopkins University (protocol number IRB00027183). All study participants gave written informed consent before their inclusion in this study.

### Role of the funders

The funders had no role in the study design, data collection, data analyses, interpretation, or writing of this manuscript.

## Results

### mRNA vaccination induces novel TCR Vβ clonotypes

To test the hypothesis that vaccination of COVID-19 convalescent patients (CCPs) would induce a repertoire of spike-specific CD4+ T cells distinct from those circulating after natural infection, we tested for T cell responses at a median of 101 (range: 35–161) days after resolution of COVID symptoms and at a median of 72 days (range: 14–241) after the second dose of either the mRNA-1273 [Moderna] or the BNT 162b2 [Pfizer-BioNTech] vaccines in 10 CCPs. PBMC were cultured for 10 days with peptide pools spanning the entirety of the spike glycoprotein of SARS-CoV-2 and three CCCs (HCoV-NL63, HCoV-229E, HCoV-OC43) (Figure 1). We then performed quantitative TCR-sequencing of the CDR3 Vβ chain of isolated CD4+ T cells to assess functional clonal expansion in response to peptide stimulation. Each peptide condition was performed in biological triplicate or duplicate wells. Using our stringent data-driven statistical algorithm alongside replicate analysis,^12^^,^^16^^,^^17^ we identified SARS-CoV-2 spike-specific memory CD4+ TCRs present after COVID-19 and after mRNA vaccination in all CCPs tested (*n* = 10, Figure S3, Table S1). To rule out the possibility that the expanded clones were non-specifically proliferating in response to cytokine production by other antigen-stimulated cells, we compared expanded clones found in the SARS-CoV-2 S stimulated wells and the positive control (CEF) stimulated wells and show there is no overlap (Figure S1) in expanded clones, indicating cytokine production is unlikely to result in non-specific expansions that would still be detected using our rigorous analytical platform. These data support use of this method to identify antigen-specific, rather than bystander, T cells. To determine the reproducibility of the assay over multiple time points. we performed two cultures from a VR a week apart and show a significant correlation in the frequency and makeup of all cells detected after the 10-day stimulation (*p* = 0.1862; Figure S2).

**Figure 2:**
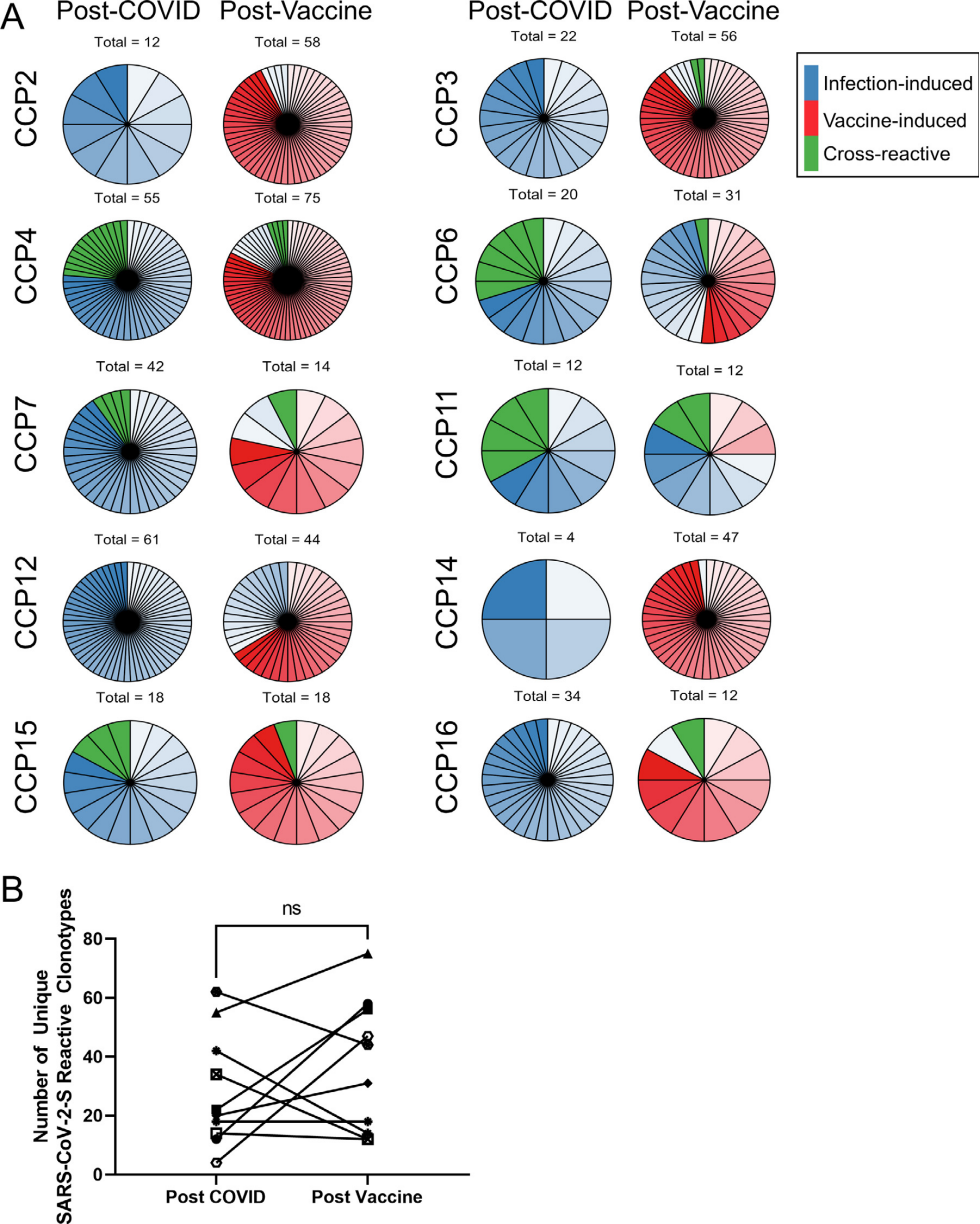
Vaccination stimulates proliferation of new SARS-CoV-2 reactive CD4+ T cells and maintains the SARS-CoV-2 repertoire. The total number of unique SARS-CoV-2 reactive clonotypes after natural infection and after vaccination are shown as pie charts for each CCP. Clonal groups are natural infection-induced (blue), vaccine-induced (red), and cross-reactive (green). Each unique clonotype is represented by a distinct shade of color and section of chart. (a). These data are further quantified with each paired data point representing the total number of unique clones in each patient at both timepoints (b). Wilcoxon signed-rank test was performed to compare the number of CD4+ T cell clonotypes after vaccination to the number of clonotypes after natural infection. ns: p ≥ 0.05.

Recent studies have shown that SARS-CoV-2-specific CD4+ T cells persist for up to 6 months after infection or vaccination.^27^, ^28^, ^29^, ^30^ Thus, we sought to test whether infection-induced clones endure or if vaccination is necessary to maintain a repertoire of SARS-CoV-2 reactive T cells. The post-vaccine timepoint was at a median of 219 days after COVID-19 disease (range: 88–455: Table S2). The median number of unique SARS-CoV-2 CD4+ T cell clonotypes increased from 21 (range: 4–62; Table S2) after natural infection to 38 (range: 12–75) after vaccination (Figure 2a). There was no significant change in the total number of reactive clonotypes between post-infection and post-vaccination (*p* = 0.4258 [Wilcoxon signed rank test]; Figure 2b). However, six of ten CCPs (60%) had a greater number of SARS-CoV-2-reactive clonotypes after vaccination than after COVID-19 (Figure 2a, b).

**Figure 3:**
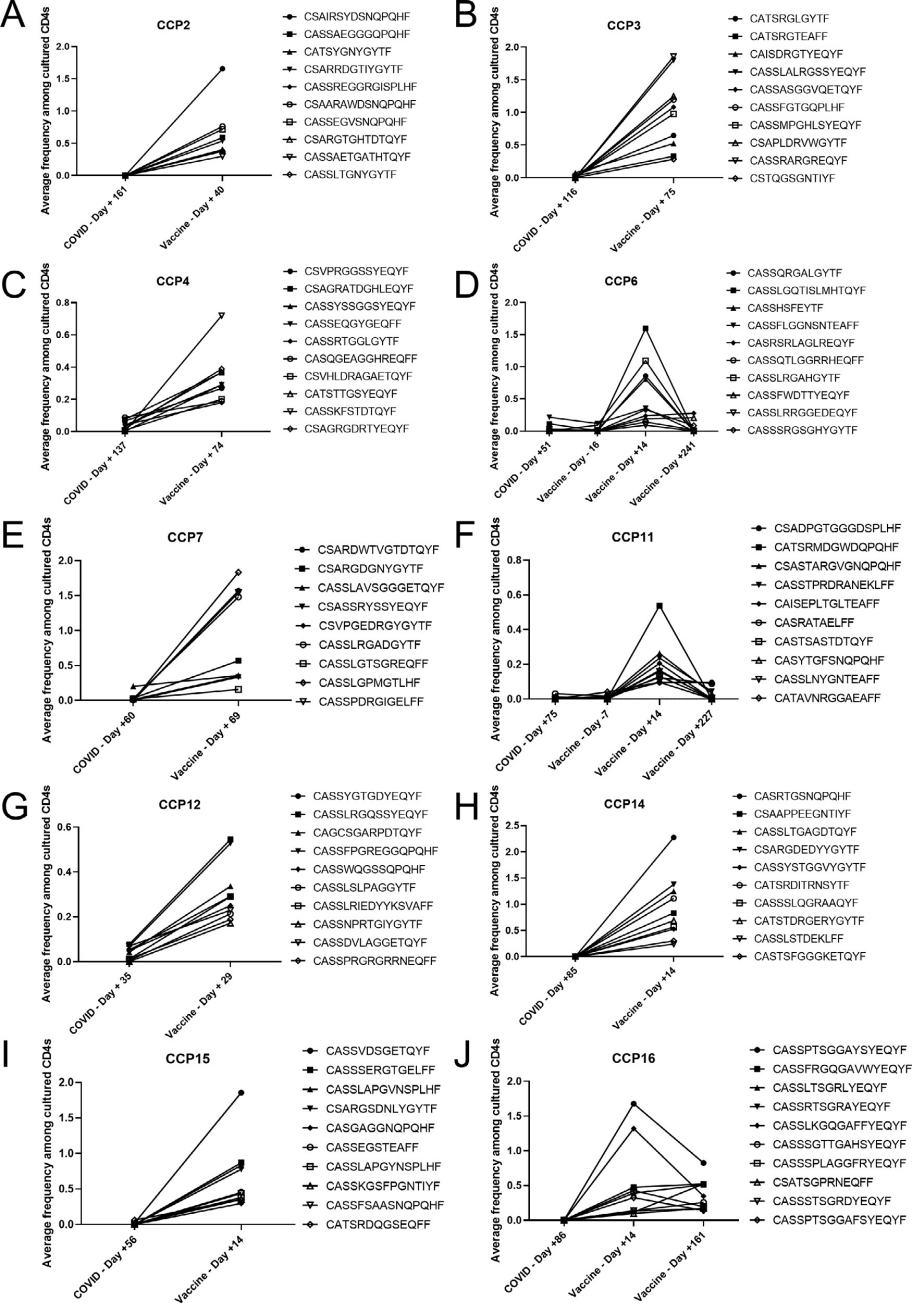
Vaccination induces activation and expansion of novel vaccine-specific clonotypes. The average frequency between each SARS-CoV-2 spike protein pool triplicate of the top 10 significantly (FDR <0.05) expanded vaccine-induced SARS-CoV-2-reactive TCRs from the earliest post-vaccine timepoint were plotted with frequency among cultured CD4+ T cells on y-axis and time point of SARS-CoV-2 spike peptide pool stimulation on x-axis (COVID = days relative to SARS-CoV-2 infection, Vaccine = days relative to vaccination). All 9 vaccine-induced clones are shown for CCP7 (e). Pre-vaccine timepoint was done for CCP6 (d) and CCP11 (f). Late post-vaccine timepoint was done for CCP6 (d), CCP11 (f), and CCP16 (j).

We performed detailed analysis on unique clonotypes that expanded at the post-vaccine timepoint to understand whether the increase in the total number of clones seen in most patients was due to maintenance and re-stimulation of infection-induced clones or accumulation of new vaccine-specific clonotypes. We found that there are three classes of clonotypes: those that expanded after COVID-19 disease (Figure 2a, blue), SARS-CoV-2/CCC spike glycoprotein cross-reactive clonotypes that may have been primed by SARS-CoV-2 infection or vaccination or by an endemic CCC (Figure 2a, green), and a distinct set of new SARS-CoV-2 mono-reactive clones that are only detected after vaccination (Figures 2a, red; 3). We plotted the average frequency in the SARS-CoV-2 triplicate wells of the most frequent novel clones found at the earliest post-vaccine time point a median of 22 days post vaccine (range: 14-75) and compared this to their average frequency at all additional timepoints studied. All CCPs studied demonstrated this vaccine-specific response (Figure 3). Of note, CCP6, CCP11, and CCP16, on whom we evaluated a late post vaccine timepoint, exhibited clonal contraction at this late phase 241-, 227-, and 161-days post vaccine, respectively.

There were also three distinct patterns of clonal expansion and contraction: (1) vaccine-induced (only detected after vaccination), (2) infection-induced with persistence through the post-vaccine timepoint, and (3) infection-induced with disappearance by the post-vaccine timepoint. We show the average frequency in the SARS-CoV-2 triplicate wells of the top 10 most frequent COVID-19 infection-induced clones and compared this to their average expansion frequency at all other timepoints (Figures S3, S4). Of the total clones expanded at the post-vaccine timepoint, a median of 10.2% (range: 0%–58.3%) were also detected prior to vaccination and were mono-reactive for SARS-CoV-2, suggesting that these clones were likely primed during SARS-CoV-2 infection. Notably, a remaining 86.3% (range: 25%–97.9%) of the clones were only detected following vaccination and were also mono-reactive for SARS-CoV-2, suggesting that these clones were specifically primed by vaccination. Only 18.7% (range: 0%–33.3%) of clones detected post COVID-19 disease persisted until after vaccination, with the remaining clones contracting below detectable levels. We also performed the ViraFEST assay on cells isolated from CCP6 and CCP11 at a pre-vaccine timepoint 16 days and one week prior to the first vaccine dose and 142 and 148 days, respectively, after their COVID-19 diagnosis (Figure 3d, f). The vaccine-induced clones were also undetectable at this pre-vaccine timepoint indicating they are likely not the result of undocumented SARS-CoV-2 re-infection between the known COVID-19 diagnosis and vaccination. This response reveals new polyclonal reactivity to vaccine epitopes not detected during natural infection. These findings suggest that the vaccine may differ from natural infection in either the breadth of epitopes presented, or in the dominant epitopes synthesized that result in activation and expansion of novel T cells. To directly test these hypotheses, we performed ELISpot assays from new healthy vaccinated individuals before and after they received booster vaccination. We found that both patients had a broadened immune response after boost resulting in a larger number of SARS-CoV-2 spike epitopes recognized (Table S3). We then carried out *ex-vivo* TCR-sequencing on uncultured PBMC from these individuals and evaluated both the circulating frequency of the SARS-CoV-2 spike-reactive clonotypes as defined by the FEST assay and the total number of unique clonotypes detected in the peripheral blood. We show that after boost, the circulating frequency of the SARS-CoV-2-reactive clones significantly increased for both VRs (Figure S5a, c; VR8: *p* = 0.0035; VR10: *p* < 0.0001 [Wilcoxon signed rank test]). Additionally, the number of unique clonotypes also increased for both patients (Figure S5b, d) resulting in both persistent diversification of the reactive repertoire and increased circulating frequency of these clones. These data demonstrate that vaccination following natural infection induces a T cell repertoire that is distinct from the repertoire induced by natural infection alone and that this distinct repertoire is correlated with diversification of the SARS-CoV-2 spike epitopes recognized

### Vaccination preferentially stimulates mono-reactive CD4+ clonotypes

**Figure 4:**
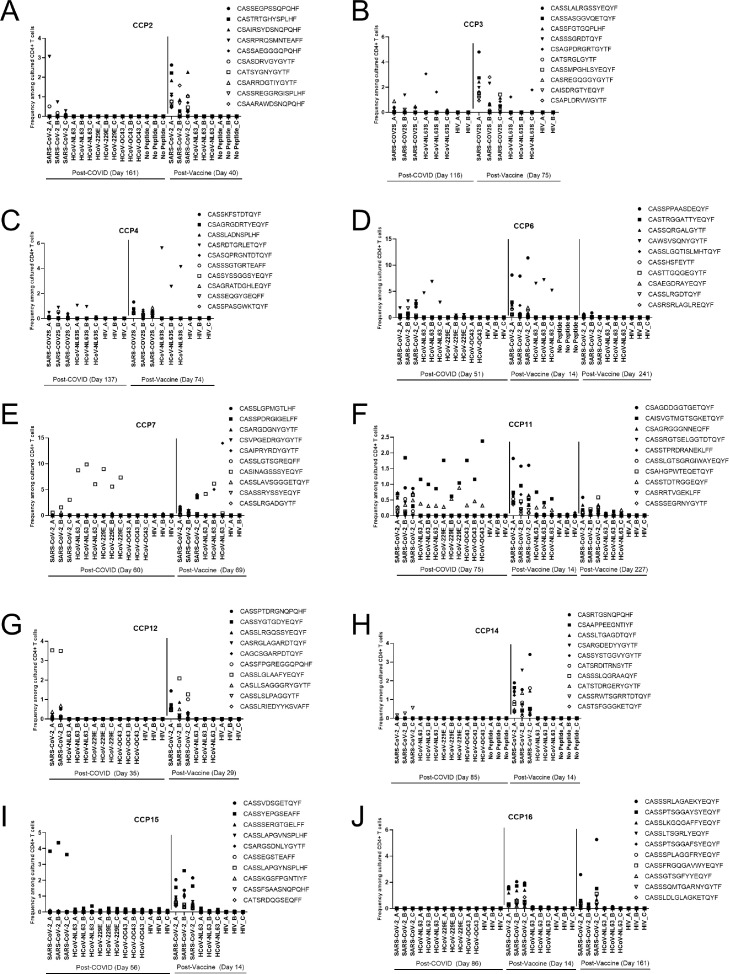
COVID-19 convalescent patients mount mono-reactive SARS-CoV-2 S CD4+ T cell responses after vaccination. FEST assays were performed on PBMC samples from study participants prior to and following SARS-CoV-2 mRNA vaccination. The top 10 significantly (FDR <0.05) expanded SARS-CoV-2 specific CD4+ T cell clonotypes at post vaccine timepoint are shown from patients with history of COVID-19 at post-COVID, post-vaccine, and late post-vaccine (e, f, j) timepoints. Frequency among cultured CD4+ T cells is shown on y-axis and each replicate of the peptide pool or control conditions are shown on x-axis. Each TCR clonotypes is represented by a unique symbol.

We next asked whether the mRNA vaccines preferentially amplify less-avid T cell clonotypes, such as those that cross-recognize a CCC and SARS-CoV-2, or more avid mono-reactive clonotypes, that specifically expand in response to SARS-CoV-2 alone.^12^ We quantifiably ranked the SARS-CoV-2-reactive clones expanding at the post vaccine timepoint using their relative frequency within each peptide-stimulated condition and determined which of these clones were cross-reactive or mono-reactive in CCPs (*n* = 10; Figure 4) and healthy vaccine recipients (VRs, *n* = 4; Figure S4). The post-culture frequency of the top 10 most frequent clones at the post-vaccine timepoint and the post-COVID timepoint is shown for each patient (Figures 4 and S3). These clones were also classified as either mono-reactive (recognizing only SARS-CoV-2) or cross-reactive (recognizing SARS-CoV-2 and the spike glycoprotein from at least one other CCC).

**Figure 5:**
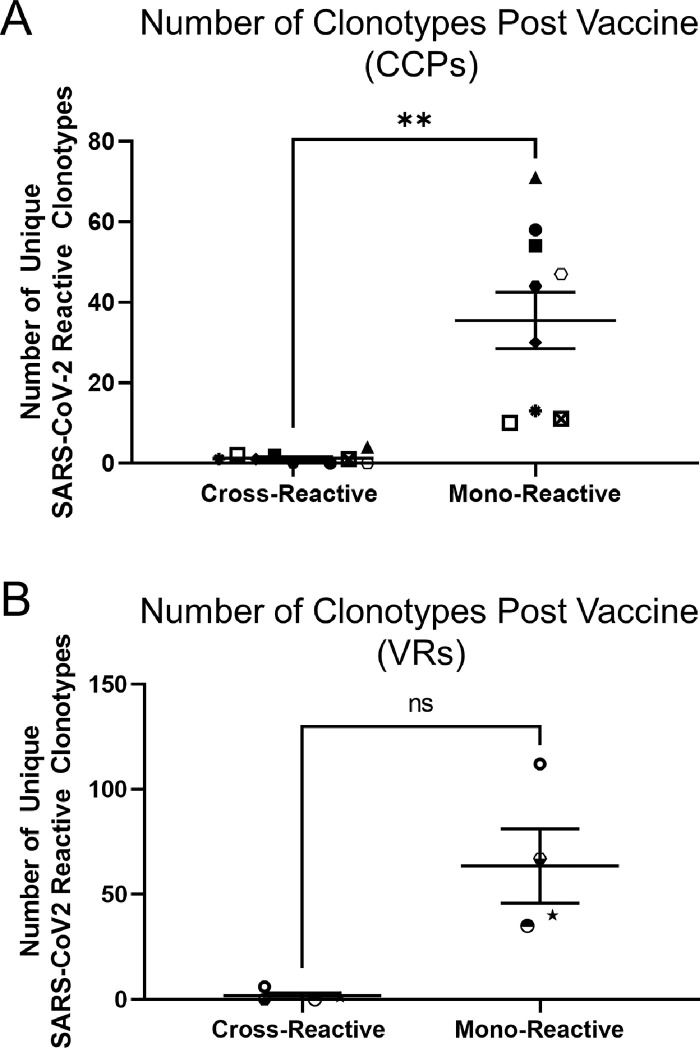
SARS-CoV-2 vaccination preferentially stimulates mono-reactive T cell responses. The absolute number of mono- and cross-reactive clonotypes are shown for CCPs (a) and VRs (b). Each patient is represented by a unique symbol. Wilcoxon signed-rank test was performed to compare the total number of unique mono- and cross-reactive clonotypes after vaccination for both CCPs and VRs. **: *p* < 0.01; ns: *p* > 0.05; non-significant.

Remarkably 304 of the 307 total (99%) CCP vaccine-specific clones were mono-reactive for SARS-CoV-2 spike peptides. In contrast, 33 of the 376 (8.78%) clones present post COVID-19 disease were cross-reactive for SARS-CoV-2 and at least one other CCC; with 5 of 10 (50%) CCPs having at least one cross-reactive clone at the post COVID timepoint (range: 3–13 unique clones). Nine of these clones persisted through the post-vaccine timepoint. CCP3 had two cross-reactive clonotypes that were only detected post vaccination and CCP16 had one cross-reactive clonotype only detected after vaccination. These data indicate that, while vaccination has the potential to induce cross-reactive responses, these clones represent a small minority of the total SARS-CoV-2-specific T cell repertoire. They further indicate that cross-reactive clones contribute to the overall SARS-CoV-2-reactive repertoire through their persistence over time, even if they do not represent a significant proportion of the vaccine-induced population. Overall, significantly fewer cross-reactive T cells were induced by vaccination relative to the number of mono-reactive T cells in CCPs (*p* = 0.0020; Figure 5a).

To assess whether this largely mono-reactive response is due to an underlying immunologic mechanism in subjects with prior COVID-19 disease, we also evaluated the vaccine-specific repertoire in healthy vaccine recipients (VRs) with no history of SARS-CoV-2 infection as documented by negative SARS-CoV-2 nucleocapsid antibody test at the time of blood draw. Only 1 in 255 (0.39%) total vaccine-induced clones were cross-reactive in VRs. Seven cross-reactive clones persisted from the pre-vaccine timepoint to the post-vaccine timepoint together making up 2.67% (7 of 262) of the total SARS-CoV-2-reactive repertoire post-vaccination. Thus, the trend of preferential stimulation of mono-reactive T cells was also observed in vaccine recipients (*p* = 0.1250 [Wilcoxon signed rank test]; Figure 5b).

To understand the endogenous frequency of SARS-CoV-2-reactive T cells, we performed *ex vivo* bulk TCR-sequencing on uncultured CD4+ T cells for CCP11 at each timepoint and used the TCR sequences as a “barcode” to track the frequency of these clones in uncultured peripheral blood over time. Mono-reactive clones that were initially primed by natural SARS-CoV-2 infection significantly expanded in the peripheral blood immediately following vaccination (*p* < 0.001 [Wilcoxon signed rank test]; Figure S6a), whereas cross-reactive clones did not change in their circulating frequency (*p* = 0.625 [Wilcoxon signed rank test]; Figure S6b). This transient expansion of mono-reactive clones was followed by a return to pre-vaccine frequency ∼7 months post-vaccination. As expected, the vaccine-induced clones were significantly expanded immediately following vaccination (*p* < 0.0001 [Wilcoxon signed rank test]; Figure S6c). Although these data are from a single participant, they support the conclusions that vaccination preferentially stimulates SARS-CoV-2 mono-reactive T cells both among novel vaccine-induced clones and re-stimulated mono-reactive clones persisting from infection.

We next generated unrooted phylogenic trees to test if there was group-specific TCR Vβ CDR3 sequence homology that could distinguish vaccine-induced vs. infection-induced clones. Preferential motif sharing, a surrogate for shared antigen recognition, may imply separate epitope processing and presentation between infection and vaccination. In five CCPs, all clones were interspersed without any preferential grouping by motif (Figure S7c-g), suggesting a diverse T cell response against many different spike epitopes. However, in CCP2, CCP3, and CCP16, there was some preferential grouping of vaccine-induced clones away from infection-induced clones, indicating there may be differential dominant antigens recognized during infection vs. vaccination in some individuals (Figure S7a, b, j).

## Discussion

There are several studies that have directly linked TCR repertoire diversity to better clinical outcomes. Broad, polyclonal anti-viral T cell responses are associated with protection from pathogens,^31^ effective responses to cancer immunotherapy,^32^ and polyfunctional immune responses against SARS-CoV-2.^33^ TCR repertoire diversity, especially in the case of CD4+ T cells that are largely polyfunctional, can lead to a variety of immunologic roles formed and overall, a more effective immune response. As an example, Panagioti et al., showed that not all antigen specific CD4+ T cell share the same functionality and ability to become long-term memory cells. Thus, it is necessary for a polyclonal immune response against CMV to first clear then virus via Th1-mediated IFN-gamma, and then provide protective immunity through durable memory responses resulting in long-term antibody production.^34^ In our study, we demonstrate that vaccination preferentially induces monospecific SARS-CoV-2 reactive T cells, which are associated with higher functional avidity.^10^^,^^12^ While we and others have shown enhancement in CCC-reactive responses post SARS-CoV-2 vaccination^35^, ^36^, ^37^, ^38^ the magnitude of the response does not approach that of SARS-CoV-2 spike peptides, which is consistent with the induction of a predominantly monospecific response to SARS-CoV-2. Given that high avidity TCRs are associated with more effective viral elimination,^39^, ^40^, ^41^ these data support vaccination as a necessary approach for generation of a long-lived and diverse repertoire of SARS-CoV-2-reactive T cells, even in patients with a prior history of COVID-19 disease. Additionally, we show that at the late post vaccination time point all patients studied had remarkable clonal contraction of both the infection-induced and vaccine-induced clones. These data further the support the waning of T cell responses over time. Future work should evaluate the long-term duration of vaccine-induced immunity, particularly in the context of booster and hybrid vaccination.

It is unclear why vaccination may result in a divergent TCR repertoire when compared to that induced by natural infection. It has been shown that mRNA vaccination induces spike-specific T cells that recognize a variety of spike epitopes and are highly activated with production of IL-2 and IFN-γ, consistent with and Th1 memory responses effective against virally infected cells.^42^ Unlike natural infection, which requires endogenous or exogenous processing of multiple viral proteins, mRNA vaccination provides an optimized mRNA transcript for efficient translation into the spike glycoprotein. There have a been a multitude of recent advances in mRNA vaccine technology which allows for the synthetic mRNA to be highly translatable and results in prolonged antigen expression *in vivo* at high concentrations.^43^ Additionally, mRNA itself is inherently immunostimulatory^44^ and thus providing a large dose of mRNA in a localized region may result in adjuvant activation of the innate immune system which could lead to enhanced dendritic cell maturation and thus T cell immune responses. We believe that together, the optimized formulation of the mRNA vaccines and the mono-antigen approach, leads to activation of a unique T cell repertoire that is distinct from that activated during natural infection. Furthermore, the optimized spike transcript and possible unique activation of innate sensing molecules may lead to different spike epitopes being effectively presented on MHC class II molecules. Importantly, the mono-antigen approach likely decreases the effect of epitope competition from nucleocapsid and nonstructural epitopes, thus having the potential to stimulate a distinct vaccine-induced repertoire.

Our study is limited by the relatively small number of CCPs studied and we recognize that, due to restriction in sample collection from non-hospitalized SARS-CoV-2 infected individuals, we did not analyze early time points following natural infection. Despite this, our comprehensive analysis of the SARS-CoV-2 reactive T cell repertoire in vaccinated patients with and without prior COVID-19 disease provide the foundation for follow-up studies to study this intriguing phenomenon more systematically. Importantly, our study suggests that vaccination following infection may induce a repertoire that is distinct from that induced by natural infection alone. Thus, our data serve as a proof-of-principle to demonstrate the increased immunologic benefit of vaccination in induction of high avidity mono-reactive T cell responses following SARS-CoV-2 mRNA vaccination. Importantly, the differences in the SARS-CoV-2-specific T cell repertoire observed after vaccination may have major implications in our understanding of correlates of protective immunity to SARS-CoV-2 and in the design of future vaccines.

## Contributors

AGD performed the experiments and acquired the data. BAW, CCG, LSC, and JXC assisted with sample collection and processing and experimental components. RR, TW, and LZ performed TCR sequencing and data pre-processing, BZ, HJ, and JZ led the bioinformatic and computational analyses. KNS and JNB led study design and data interpretation. DMP and ALC assisted with study design, data interpretation, and manuscript planning. AGD, BAW, BZ, KNS and JNB verified the underlying data. AGD drafted the manuscript. All authors contributed to experimental planning, data analysis and interpretation, and manuscript preparation. All authors have read and approved the final and submitted version of the manuscript.

## Data sharing statements

All raw TCR sequencing data has been made publicly available. Illumina data has been uploaded to SRA under the BioProject accession: PRJNA705196 CD4+ T Cell Receptor Sequencing of COVID-19 Convalescent, Vaccinated, or Pre-COVID Healthy Donors. CCP6 pre- and post-vaccine timepoints, and healthy vaccine recipients, VR8, VR10, and VR14 were sequenced using the Adaptive Biotechnologies TCR-sequencing kit. These data can be accessed here: DOI- 10.21417/AGD2022EBM and the URL- https://clients.adaptivebiotech.com/pub/dykema-2022-ebiomed. All processed FEST data with subsequent analyses has been attached to this publication as a supplemental excel document.

## Declaration of interests

Dr. Smith reports grants from NIH, during the conduct of the study; In addition, Dr. Smith has a patent US provisional patent application no. 62/407,820 pending, and a patent US provisional patent application no. 63/135,534 pending and Stock options and ownership equity: ManaT Bio, IncÜhair, SITC Big Data and Data Sharing, Committee Honoraria for presentations: Adaptive Biotechnologies, Honoraria for presentations: Illumina Inc. Dr. Blankson has a patent US provisional patent application no. 63/135,534 pending. Dr. Cox reports grants from NIH, during the conduct of the study; in addition, Dr. Cox has a patent US provisional patent application no. 62/135,534 pending. Dr. Pardoll has a patent US provisional patent application no. 62/135,534 pending. A. Dykema has a patent US provisional patent application no. 62/135,534 pending. These arrangements have been reviewed and approved by the Johns Hopkins University in accordance with its conflict-of-interest policies.

## Appendix Supplementary materials

Image, application 1

Image, application 2
