# Supplementary material for: SARS-CoV-2 vaccination diversifies the CD4+ spike-reactive T cell repertoire in patients with prior SARS-CoV-2 infection
Source: eBioMedicine. 2022 May 6;80:104048. doi: 10.1016/j.ebiom.2022.104048 (PMC9073272; doi:10.1016/j.ebiom.2022.104048)
Supplement: Supplementary file 2 [file mmc2.docx]

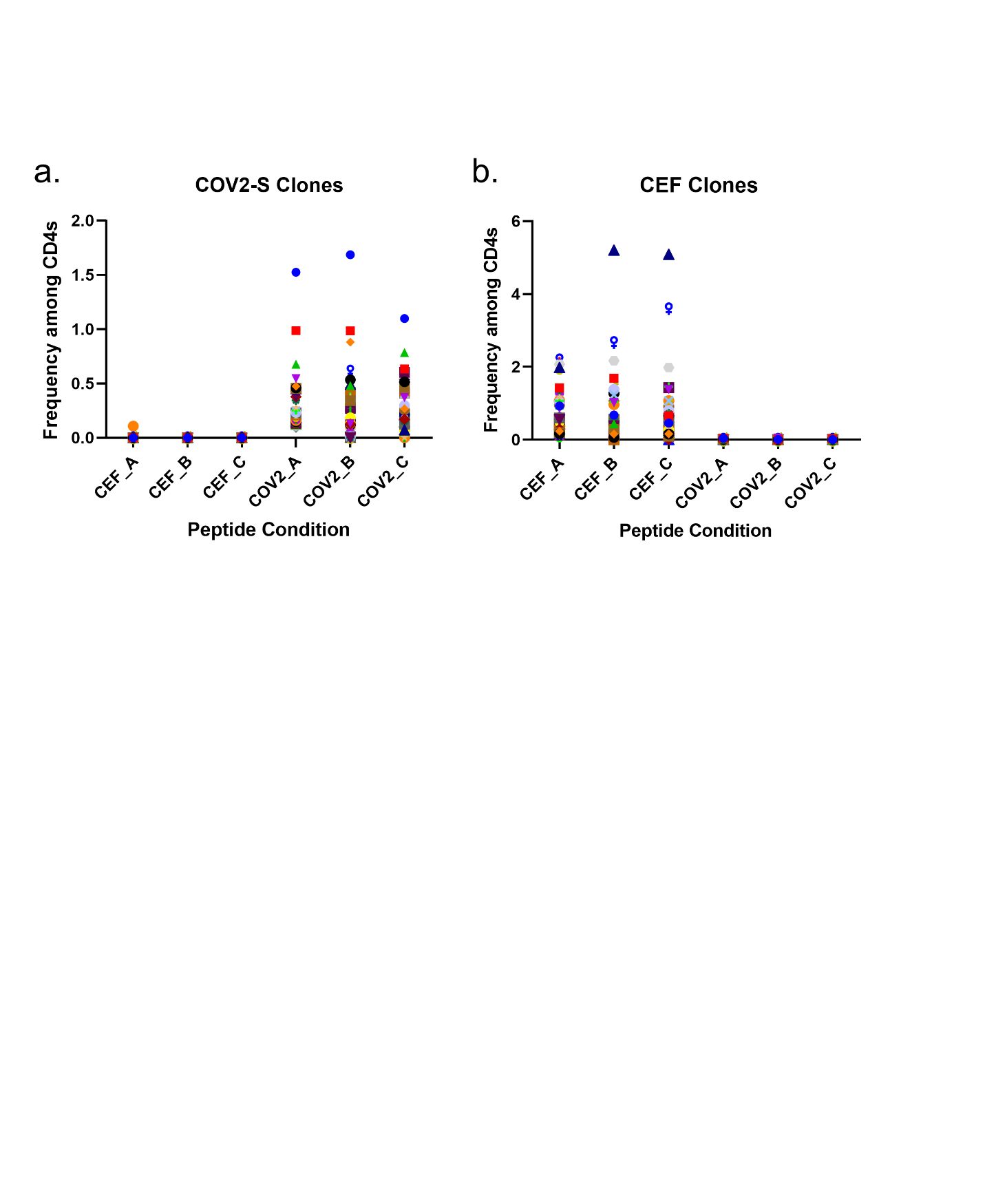


**Figure S1: SARS-CoV-2 S-reactive clonotypes expand in an antigen-dependent manner.** FEST assays were performed on PBMC samples from all CCPs. All SARS-CoV-2 **(a)** and CMV, EBV, Flu (CEF; **b**)-reactive clonotypes are shown for SARS-CoV-2 and CEF positive control wells from a representative patient. Each unique clone is represented by a distinct color/symbol.


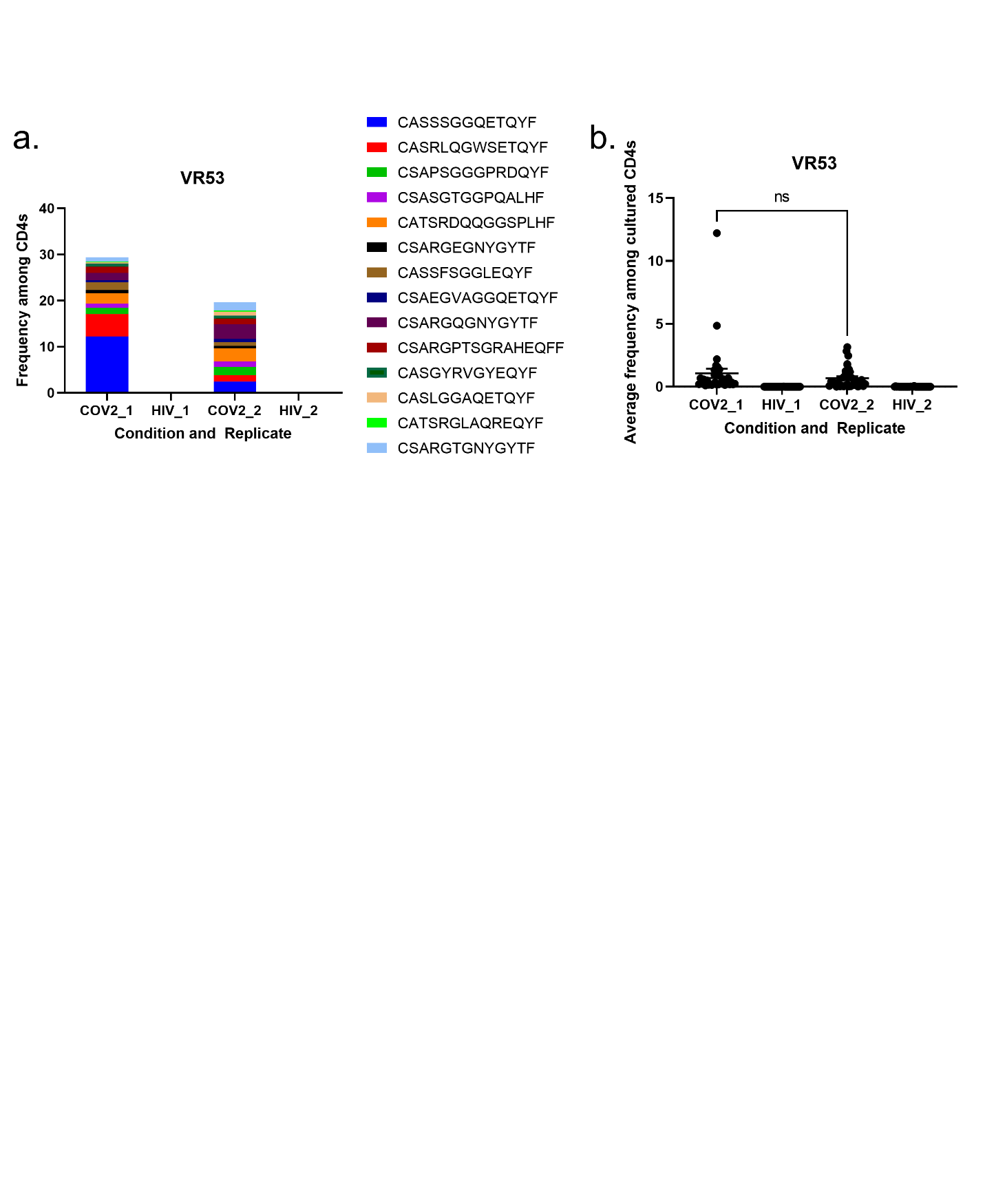


**Figure S2: FEST assay reproducibly determines SARS-CoV-2 reactive CD4+ clonotypes across distinct blood draw timepoints.** PBMC from a healthy vaccinated donor (VR53) from two blood draw timepoints one week apart (Replicate 1 and Replicate 2) were stimulated with our SARS-CoV-2 spike glycoprotein pool as well as the HIV negative control. The averaged frequency between replicate wells of the top 10 expanded clonotypes at each time point (clones present in the top 10 of both timepoints are only shown once) are shown (**a**). The average frequency of all positive clones (each point represents a unique clonotype) at each FEST time point are shown (Wilcoxon signed rank test, ns: p>0.05; **b**).


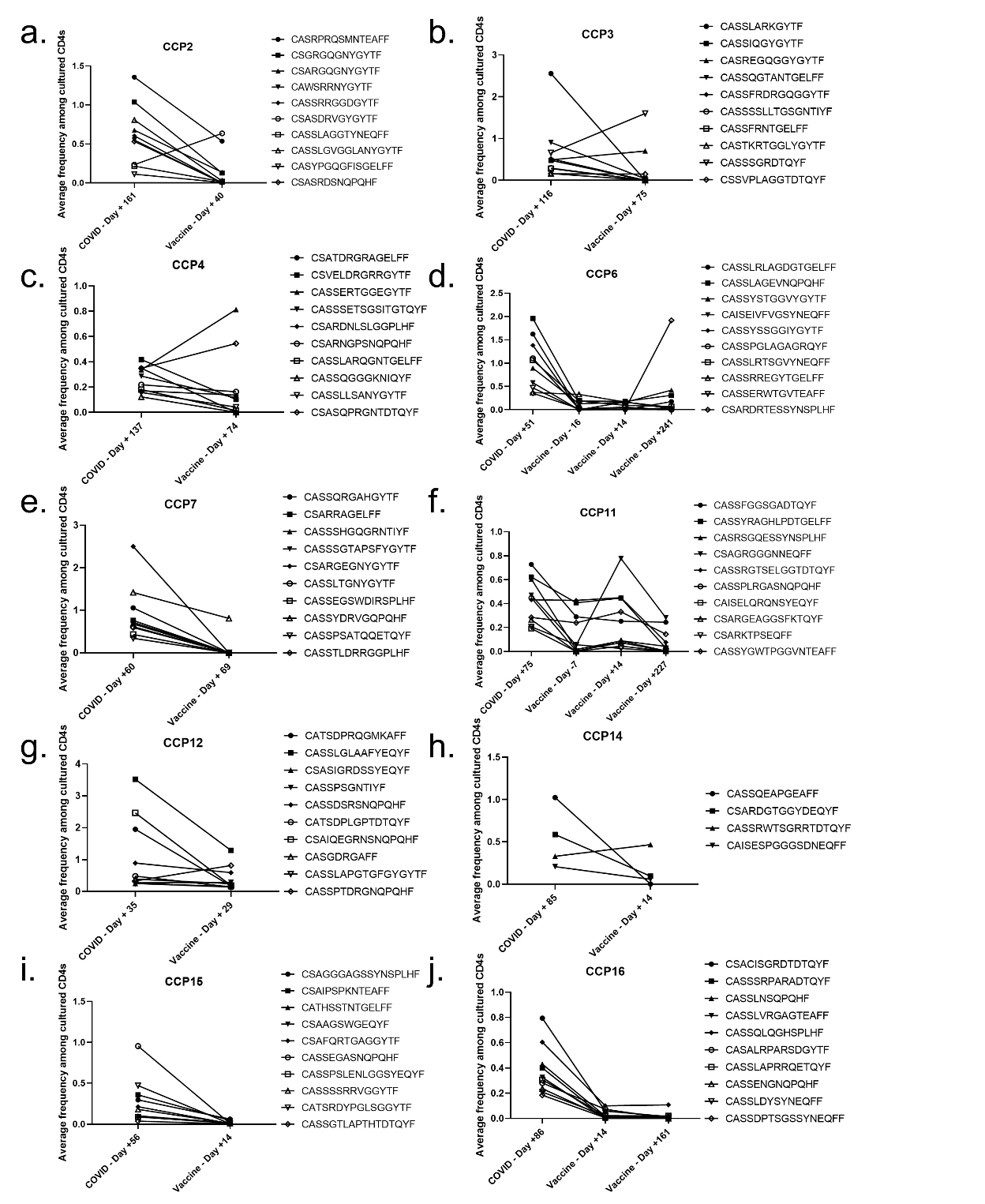


**Figure S3: COVID-19-induced clonotypes contract overtime.** The average frequency between each SARS-CoV-2 spike protein pool triplicate of the top 10 significantly (FDR <0.05) expanded COVID-induced SARS-CoV-2-reactive TCRs were plotted with frequency among cultured CD4+ T cells on y-axis and time point of SARS-CoV-2 spike peptide pool stimulation on x-axis (COVID = days relative to SARS-CoV-2 infection, Vaccine = days relative to vaccination). All 4 COVID-induced clones are shown for CCP14 **(c).** Pre-vaccine timepoint was done for CCP6 (**b)** and CCP11 **(d).** Late post-vaccine timepoint was done for CCP6 (**b)**, CCP11 **(d)**, and CCP16 (**j**).


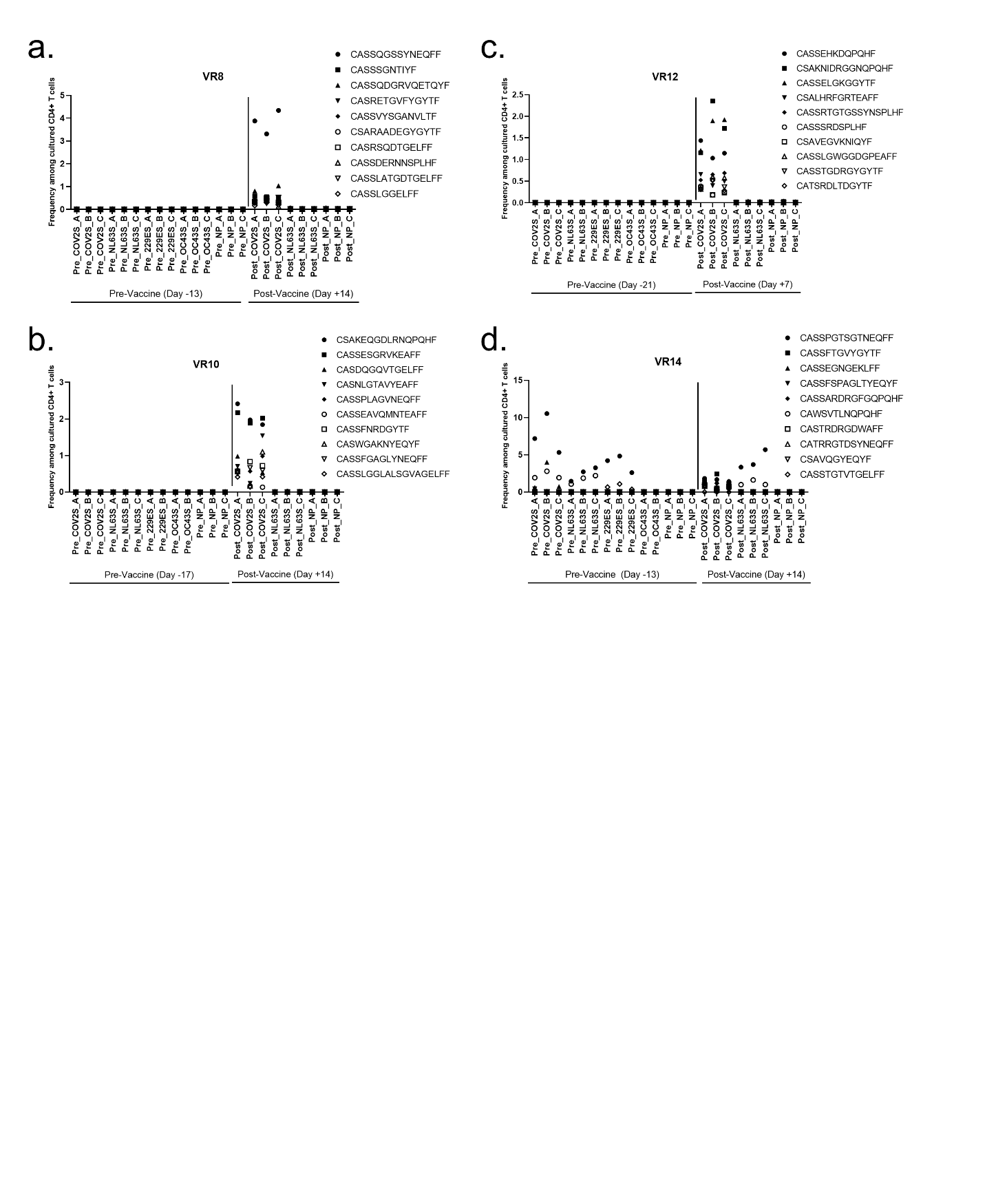


**Figure S4: Mono-reactive responses dominant in healthy vaccine recipients and increase in frequency in peripheral blood after vaccination.** FEST assays were performed on PBMC samples from healthy vaccine recipients (VRs) prior to and after SARS-CoV-2 mRNA vaccination. The top 10 significantly (FDR <0.05) expanded SARS-CoV-2 specific CD4+ T cell clonotypes at post vaccine timepoint are shown from patients without history of COVID-19 (**a-d).**


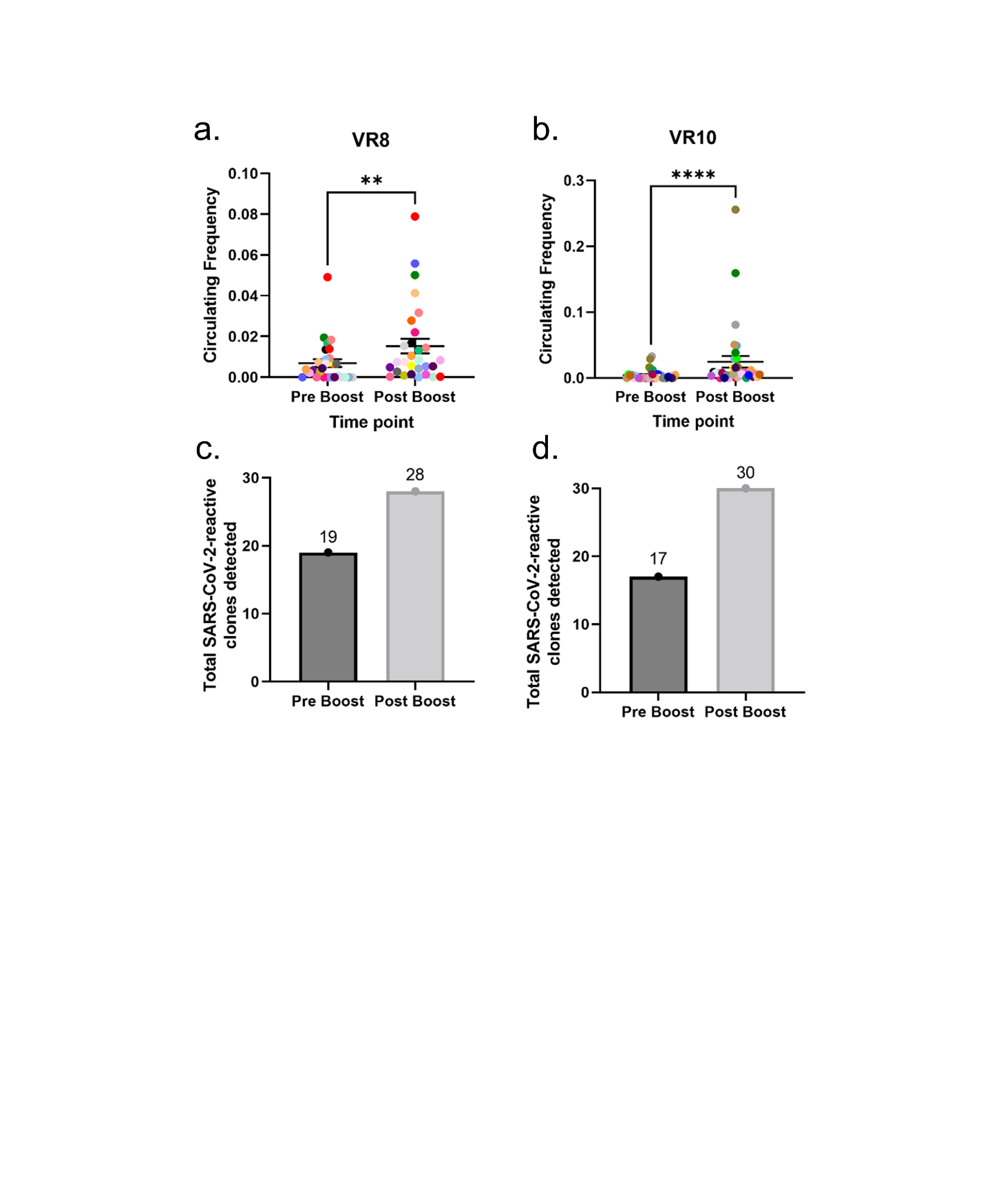


**Figure S5: Vaccination increases both circulating frequency and number of unique SARS-CoV-2-reactive clonotypes.** Ex vivo circulating frequency and absolute number of SARS-CoV-2 vaccine-induced clones are shown for two healthy vaccine recipients: VR8 (**a, c**), and VR10 **(b, d)** pre and post SARS-CoV-2 mRNA vaccine booster. Each paired TCR clonotype is represented by a distinct color on the dot plot. **: p =0.0035, ****: p <0.0001. Wilcoxon signed rank test was used to compare the frequency of clonotypes between timepoints.


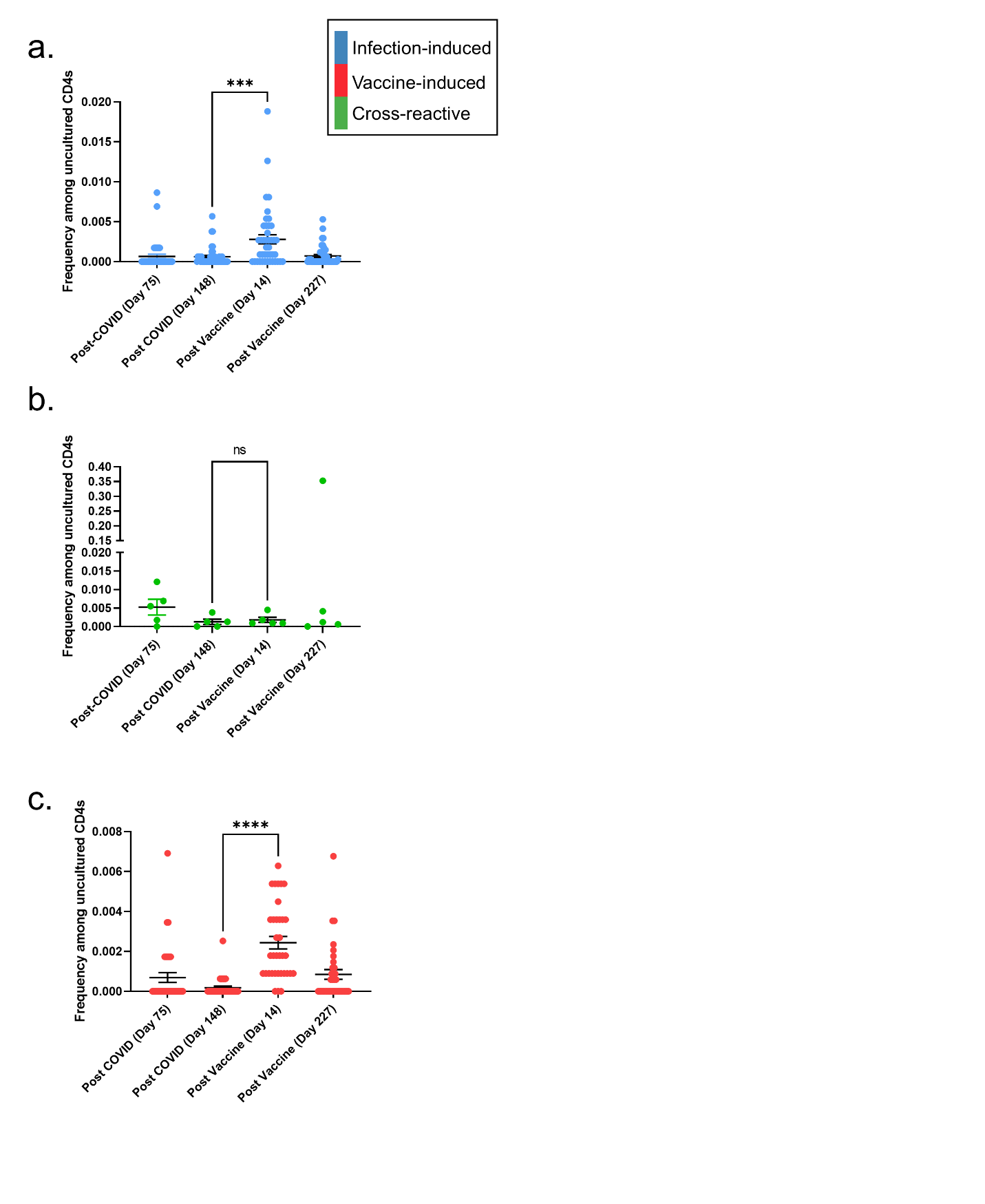


**Figure S6: Mono-reactive T cells increase in frequency in peripheral blood after vaccination.** Ex vivo circulating frequency of infection induced-mono-**(a),** cross-reactive **(b)**, and vaccine **(c)**-induced clonotypes are shown for CCP11 from TCR-seq on uncultured CD4+ T cells at each timepoint. *******: p < 0.001, ****: p<0.0001, ns: p>0.05. Wilcoxon signed rank test was used to compare the frequency of clonotypes between pre and early post vaccine timepoints.


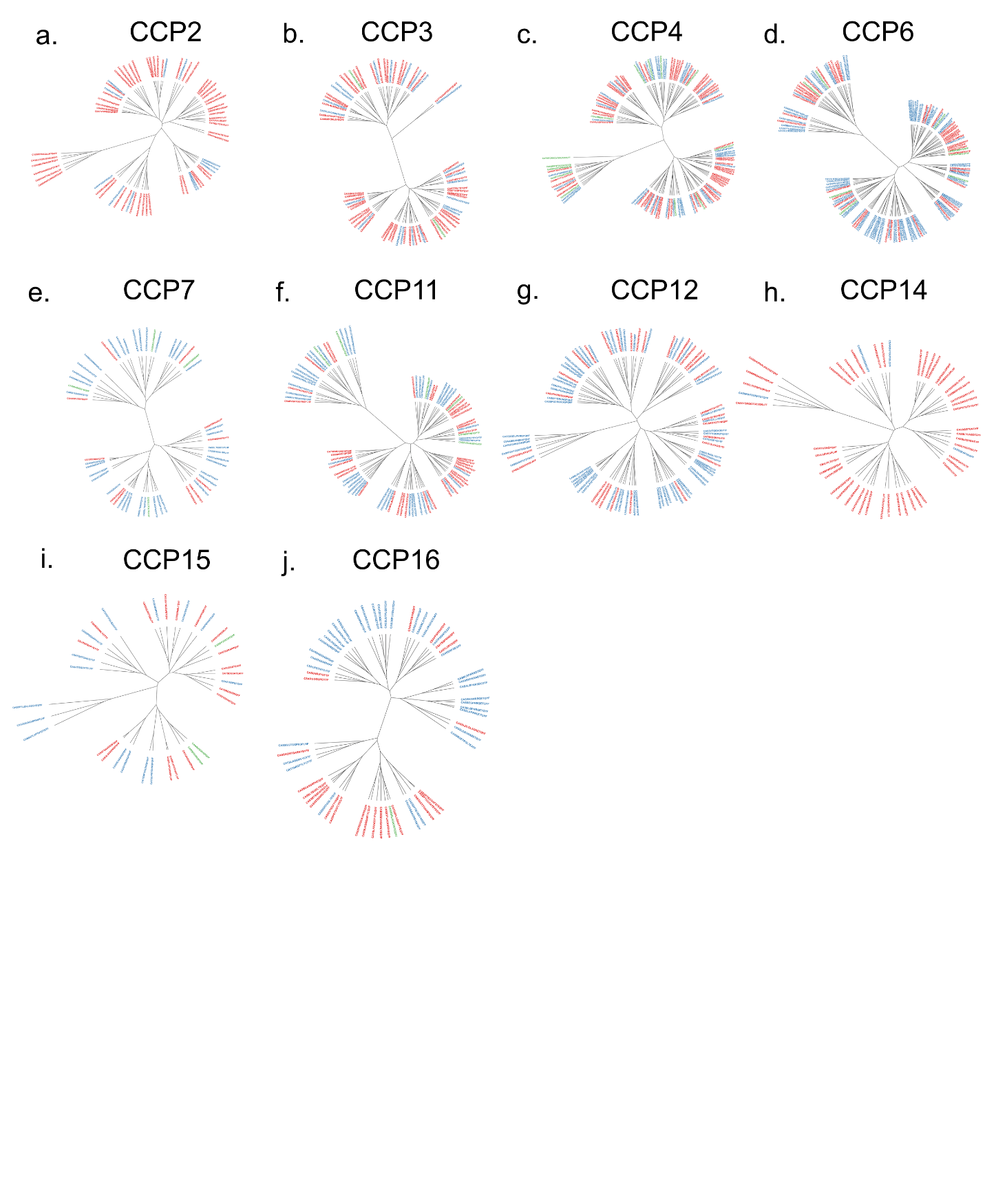


**Figure S7: COVID-19-induced and vaccine-induced clones do not cluster by conserved TCR Vβ CDR3 regions.** Unrooted phylogenetic tree for natural infection-induced (blue) vaccine-induced (red), and cross-reactive (green) clonotypes. Each leaf represents a unique clonotype.

**Table S2: Days post COVID-19 disease and SARS-CoV-2 mRNA vaccination for patient biospecimen collection.**

**Table S3: Peptides targeted by VR8 and VR10 pre-boost and post-boost doses as measured by IFN-y ELISpot assay. Spot forming units (SFU) per million PBMCs and Stimulation Index are shown for each peptide.**

| **VR8** | | | | | |
| --- | --- | --- | --- | --- | --- |
| **Pre-boost** | | | **Post-boost** | | |
| Peptide Sequence | SFU / Million PBMCs | Stimulation Index | Peptide Sequence | SFU / Million PBMCs | Stimulation Index |
| 197-IDGYFKIYSKHTPINLV-213 | 87 | 19 | 127-VIKVCEFQFCNDPFLGV-143 | 30 | 15 |
| 211-NLVRDLPQGFSALEPLV-227 | 26 | 6 | 190-REFVFKNIDGYFKIYSK-206 | 22 | 11 |
| 267-VGYLQPRTFLLKYNENG-283 | 30 | 7 | 197-IDGYFKIYSKHTPINLV-213 | 83 | 41 |
| 309-EKGIYQTSNFRVQPTES-325 | 22 | 5 | 204-YSKHTPINLVRDLPQGF-220 | 22 | 11 |
| 316-SNFRVQPTESIVRFPNI-332 | 22 | 5 | 211-NLVRDLPQGFSALEPLV-227 | 30 | 15 |
| 351-YAWNRKRISNCVADYSV-367 | 48 | 10 | 225-PLVDLPIGINITRFQTL-241 | 22 | 11 |
| 379-CYGVSPTKLNDLCFTNV-395 | 104 | 22 | 239-QTLLALHRSYLTPGDSS-255 | 78 | 39 |
| 400-FVIRGDEVRQIAPGQTG-416 | 39 | 8 | 246-RSYLTPGDSSSGWTAGA-262 | 26 | 13 |
| 491-PLQSYGFQPTNGVGYQP-507 | 30 | 7 | 267-VGYLQPRTFLLKYNENG-283 | 296 | 147 |
| 715-PTNFTISVTTEILPVSM-731 | 39 | 8 | 288-AVDCALDPLSETKCTLK-304 | 22 | 11 |
| 953-NQNAQALNTLVKQLSSN-969 | 22 | 5 | 309-EKGIYQTSNFRVQPTES-325 | 39 | 20 |
|  |  |  | 351-YAWNRKRISNCVADYSV-367 | 117 | 59 |
| Untreated Control | 5 | 1 | 358-ISNCVADYSVLYNSASF-374 | 22 | 11 |
|  |  |  | 372-ASFSTFKCYGVSPTKLN-388 | 30 | 15 |
|  |  |  | 379-CYGVSPTKLNDLCFTNV-395 | 43 | 22 |
|  |  |  | 400-FVIRGDEVRQIAPGQTG-416 | 43 | 22 |
|  |  |  | 638-TGSNVFQTRAGCLIGAE-654 | 22 | 11 |
|  |  |  | 645-TRAGCLIGAEHVNNSYE-661 | 35 | 17 |
|  |  |  | 680-SPRRARSVASQSIIAYT-696 | 43 | 22 |
|  |  |  | 687-VASQSIIAYTMSLGAEN-703 | 26 | 13 |
|  |  |  | 701-AENSVAYSNNSIAIPTN-717 | 26 | 13 |
|  |  |  | 715-PTNFTISVTTEILPVSM-731 | 48 | 24 |
|  |  |  | 743-CGDSTECSNLLLQYGSF-759 | 26 | 13 |
|  |  |  | 897-PFAMQMAYRFNGIGVTQ-913 | 35 | 17 |
|  |  |  | 953-NQNAQALNTLVKQLSSN-969 | 39 | 20 |
|  |  |  | 981-LSRLDKVEAEVQIDRLI-997 | 22 | 11 |
|  |  |  | 1023-NLAATKMSECVLGQSKR-1039 | 26 | 13 |
|  |  |  |  |  |  |
|  |  |  | Untreated Control | 2 | 1 |

| **VR10** | | | | | |
| --- | --- | --- | --- | --- | --- |
| **Pre-boost** | | | **Post-boost** | | |
| Peptide Sequence | SFU / Million PBMCs | Stimulation Index | Peptide Sequence | SFU / Million PBMCs | Stimulation Index |
| 29-TNSFTRGVYYPDKVFRS-45 | 38 | 5 | 29-TNSFTRGVYYPDKVFRS-45 | 104 | 8 |
| 442-DSKVGGNYNYLYRLFRK-458 | 29 | 4 | 36-VYYPDKVFRSSVLHSTQ-52 | 68 | 5 |
| 575-AVRDPQTLEILDITPCS-591 | 21 | 3 | 43-FRSSVLHSTQDLFLPFF-59 | 44 | 3 |
| 645-TRAGCLIGAEHVNNSYE-661 | 21 | 3 | 78-RFDNPVLPFNDGVYFAS-94 | 44 | 3 |
|  |  |  | 120-VNNATNVVIKVCEFQFC-136 | 36 | 3 |
| Untreated Control | 7 | 1 | 162-SANNCTFEYVSQPFLMD-178 | 48 | 4 |
|  |  |  | 190-REFVFKNIDGYFKIYSK-206 | 64 | 5 |
|  |  |  | 491-PLQSYGFQPTNGVGYQP-507 | 40 | 3 |
|  |  |  | 505-YQPYRVVVLSFELLHAP-521 | 80 | 6 |
|  |  |  | 512-VLSFELLHAPATVCGPK-528 | 40 | 3 |
|  |  |  | 729-VSMTKTSVDCTMYICGD-745 | 44 | 3 |
|  |  |  | 736-VDCTMYICGDSTECSNL-752 | 52 | 4 |
|  |  |  | 757-GSFCTQLNRALTGIAVE-773 | 40 | 3 |
|  |  |  | 764-NRALTGIAVEQDKNTQE-780 | 48 | 4 |
|  |  |  | 785-VKQIYKTPPIKDFGGFN-801 | 44 | 3 |
|  |  |  | 799-GFNFSQILPDPSKPSKR-815 | 52 | 4 |
|  |  |  | 806-LPDPSKPSKRSFIEDLL-822 | 48 | 4 |
|  |  |  | 813-SKRSFIEDLLFNKVTLA-829 | 100 | 8 |
|  |  |  | 820-DLLFNKVTLADAGFIKQ-836 | 44 | 3 |
|  |  |  | 827-TLADAGFIKQYGDCLGD-843 | 44 | 3 |
|  |  |  | 834-IKQYGDCLGDIAARDLI-850 | 64 | 5 |
|  |  |  | 841-LGDIAARDLICAQKFNG-857 | 64 | 5 |
|  |  |  | 848-DLICAQKFNGLTVLPPL-864 | 40 | 3 |
|  |  |  | 883-TSGWTFGAGAALQIPFA-899 | 44 | 3 |
|  |  |  | 890-AGAALQIPFAMQMAYRF-906 | 68 | 5 |
|  |  |  | 897-PFAMQMAYRFNGIGVTQ-913 | 92 | 7 |
|  |  |  | 904-YRFNGIGVTQNVLYENQ-920 | 40 | 3 |
|  |  |  | 911-VTQNVLYENQKLIANQF-927 | 72 | 6 |
|  |  |  | 918-ENQKLIANQFNSAIGKI-934 | 64 | 5 |
|  |  |  | 925-NQFNSAIGKIQDSLSST-941 | 44 | 3 |
|  |  |  | 932-GKIQDSLSSTASALGKL-948 | 48 | 4 |
|  |  |  | 953-NQNAQALNTLVKQLSSN-969 | 108 | 8 |
|  |  |  | 988-EAEVQIDRLITGRLQSL-1004 | 136 | 11 |
|  |  |  | 1002-QSLQTYVTQQLIRAAEI-1018 | 44 | 3 |
|  |  |  | 1030-SECVLGQSKRVDFCGKG-1046 | 52 | 4 |
|  |  |  | 1037-SKRVDFCGKGYHLMSFP-1053 | 40 | 3 |
|  |  |  | 1058-HGVVFLHVTYVPAQEKN-1074 | 68 | 5 |
|  |  |  | 1079-PAICHDGKAHFPREGVF-1095 | 40 | 3 |
|  |  |  |  |  |  |
|  |  |  | Untreated Control | 13 | 1 |
